# Antimicrobial Activities of Extracts and Isolated Coumarins from the Roots of Four *Ferulago* Species Growing in Turkey

**DOI:** 10.22037/ijpr.2019.1100718

**Published:** 2019

**Authors:** Songül Karakaya, Duygu Şimşek, Hilal Özbek, Zühal Güvenalp, Nurten Altanlar, Cavit Kazaz, Ceyda Sibel Kiliç

**Affiliations:** a *Department of Pharmacognosy, Faculty of Pharmacy, Atatürk University, Erzurum 25240, Turkey.*; b *Department of Pharmaceutical Microbiology, Faculty of Pharmacy, Ankara University, Ankara 06100, Turkey.*; c *Department of Chemistry, Faculty of Science, Atatürk University, Erzurum 25240, Turkey.*; d *Department of Pharmaceutical Botany, Faculty of Pharmacy, Ankara University, Ankara 06100, Turkey.*

**Keywords:** Antimicrobial, Coumarin, Ferulago, Peucedanol-2′-benzoate, Prantschimgin

## Abstract

*Ferulago* species have been utilized since ancient times as digestive, sedative, aphrodisiac, along with in salads or as a spice due to their special odors. The study reports isolation and characterization of bioactive compounds of* Ferulago pachyloba (F. pachyloba)*, *Ferulago trachycarpa (F. trachycarpa),* *Ferulago bracteata* (*F. bracteata)*, and *Ferulago blancheana* (*F. blancheana)* via bioassay guided fractionation and isolation process. The structures of compounds were elucidated by detailed spectroscopic analyses. They were also assessed for their activities at 1000-31.25 µg/mL concentrations by microbroth-dilution methods. Antimicrobial activity of aqueous, methanol extracts and dichloromethane, ethyl acetate, *n*-butanol and aqueous residue fractions of methanol extracts from aerial parts and roots of species along with isolated compounds [osthole, imperatorin, bergapten, prantschimgin, peucedanol-2′-benzoate, grandivitinol, suberosin, xanthotoxin, felamidin, marmesin, umbelliferone, ulopterol and a sterol mixture consisted of stigmasterol, *β*-sitosterol] were evaluated. Antimicrobial effect has been seen against Gram-negative, Gram-positive bacteria, and a yeast *C. albicans *at a concentration between 31.25 and 62.5 μg/mL. Especially, *C. albicans* (MIC = 31.25 μg/mL) was the most inhibited microorganism. Moreover, growth of *P. aeruginosa, B. subtilis*, *E. coli*, and *S. aureus *were inhibited at 62.5 μg/mL MIC values. Among tested samples prantschimgin and dichloromethane fraction of aerial parts from *F. pachyloba* showed the best activity against *C. albicans *(MIC = 31.25 μg/mL). However, among aqueous extracts and residue fractions, only *F. blancheana* aerial parts, *F. trachycarpa* aerial parts, and roots and *F. bracteata* roots showed activity against *C. albicans*. Among microorganisms *E. coli* was found to be the least affected.

## Introduction

Anatolia is thought to be as a gene center for *Ferulago* W. Koch. (*Apiaceae*), because 35 of the 50 taxa of this genus in the world grow up and they are known as “Çakşır, cağşır, günlükotu, kılkuyruk, kuzukemirdi, şeytanteresi, and kaya kişnişi” in Turkey (1). *F. pachyloba* (Fenzl) Boiss., *F. bracteata* Boiss. and Hausskn. and *F. blancheana *Post ex Boiss. are endemic perennial species, growing only in Niğde-Central Anatolia, Gaziantep-Southeastern Anatolia, and Kayseri-Central Anatolia, Turkey, respectively; however, *F. trachycarpa* Boiss. is not an endemic species, growing in Antalya (2, 3).

*Ferulago* species have been used in traditional medicine as carminative, sedative, vermifuge, tonic, aphrodisiac, digestive, and against hemorrhoids, ulcers, snake bites, headache, and spleen diseases, as well as salad or spice and food for goats and deers (4, 5).

Medicinal plants are conventionally utilized all over the world as remedies for the treatment of diverse diseases containing gastrointestinal symptoms, asthma, skin disorders, urinary problems and respiratory, cardiovascular, hepatic diseases. These plants synthesize a various sequence of biologically active compounds that are significant for them to survive and proceed in the natural environment, including protective functions with reference to abiotic stresses resulted from mineral nutrient, supplying water status, temperature, and to insect pests. The composition of biologically active compounds from medicinal plants changes largely depending on the plant species, on their association with microbes and soil type. These bioactive secondary metabolites synthesized by medicinal plants can also extremely influence their physiological functions and plant-associated microbial communities (6).

Previous phytochemical studies on *Ferulago *species pointed out the presence of coumarins as the most usual metabolites (7), that possess antioxidant (8, 9), antiinflammatory (10, 11), antibacterial (12), antifungal (13, 14), antiviral (15), anticancer (16, 17), anticoagulant (18, 19), anticonvulsant (20), neuroprotective (21), antiadipogenic (22), antitubercular (23), antihyperglycemic (24, 25), antihypertensive (26, 27), and antidiabetic (28, 29) activities.

There are also some reports about biological activities of some *Ferulago* species such as cytotoxic (12), acetylcholinesterase inhibitory (30), α-amylase and α-glucosidase inhibitory (31), anticoagulant (32), antimicrobial, and antioxidant (8) activities and also aphrodisiac effect on erectile dysfunction (33).

Antimicrobial resistance has being increased rapidly against current drugs during the last decades; however, new antimicrobial drug development has slow down. This situation leads health authorities to search for natural antimicrobial active substances and/or to combine them with existing approved drugs. Treatment with plants is actually a traditional method known from antic ages long before the development of modern medicine (34, 35). Antimicrobial activity of the plants comes from mostly by aromatic or phenolic substances (36). Antimicrobial substances of plants may be classified as alkaloids, essential oils, flavones, lectins, polyphenols, polypeptides, phenolics, tannins, and terpenoids (34). The current study aimed to give first report on evaluating antimicrobial activities of the extracts from root and aerial parts of *F. pachyloba*, *F. bracteata*, *F. blancheana, F. trachycarpa,* and isolated compounds.

## Experimental

*Reagents and chemicals*

Column chromatographies were performed on Silica gel 60 (0.063-0.200 mm, Merck) and Sephadex LH-20 (Fluka). TLC was carried out on pre-coated Kieselgel 60 F_254 _aluminum sheets (Merck). Mueller Hinton Broth (MHB) (Merck) for the production of single-colony bacteria and Sabouraud Dextrose Agar (SDA) (Oxoid) for the production of single colony yeast for fresh culture and in macrodilution broth were used. Stock bacterial suspensions were prepared at a density of 0.5 McFarland - with a DEN-1 densitometer (BIOSAN) device on physiological saline solution- from overnight cultures of standard strains. 

*Plant material*

Flowering plants of *F. pachyloba, F. bracteata*, *F. blancheana, *and *F. trachycarpa *were collected in 2014 from Niğde, Gaziantep, Kayseri, and Antalya (Turkey), respectively and identified by Prof. Dr. Hayri Duman, a plant taxonomist in the Department of Biology, Faculty of Science, Gazi University. The voucher specimens are kept in the Herbarium of Ankara University, Faculty of Pharmacy (Herbarium numbers are AEF 26674, AEF 26676, AEF 26673, and AEF 26677, respectively).

*Extraction and isolation*

Air-dried roots and aerial parts of *Ferulago pachyloba, F. trachycarpa, F. bracteata* and *F. blancheana* were powdered and macerated three times with methanol for 8 h in a water bath not exceeding 45 °C (4 × 2 L) using a mechanical mixer at 300 rpm, separately. Combined extracts were filtered and concentrated till dryness by rotary evaporator (Heidolph VV2000, Germany) then dispersed in methanol-water (1:9) and fractionated four times with 400 mL of dichloromethane, ethyl acetate, and *n*-butanol, respectively. The fractions were concentrated till dryness by rotary evaporator. On the other hand, 50 g of roots and aerial parts from these plants were grounded and macerated with 500 mL of distilled water for 8 h/3 days at 30 to 35 °C, separately. Aqueous extract was filtered, freezed (Sanyo Medical Freezer, Germany), and lyophilized (Christ® Gamma 2-16 LSC, Germany) to give aqueous extracts of roots and aerial parts. Amounts of the powdered plants and obtained extracts are given in Table 1. A column of 52.5 cm in length and 6.9 cm in inner diameter was used in the column chromatography.

As a result of the bioguided fractionation study, the effective dichloromethane extracts of roots from all species were first submitted to a silica gel column and eluted with a gradient of *n-*hexane:ethyl acetate (100:0 → 0:100, v/v) and ethyl acetate:methanol (100:0 → 0:100, v/v), and nine fractions (Fr. A-I) were obtained. Fr. A was subjected to a silica gel column which was eluted with a mixture of *n-*hexane:ethyl acetate (95:5) and compounds **13 **and** 14 **were obtained as a mixture. Repetitive silica gel column chromatography with *n*-hexane-ethyl acetate (90:10 and 95:5) solvent system on Fr. B gave compound **1**. Fr. C was applied to silica gel column eluting with *n-*hexane:ethyl acetate (85:15) and Sephadex LH-20 column eluting with ethyl acetate to give compounds **2** and **3**. Eluting with *n*-hexane-ethyl acetate (90:10) over silica gel column of Fr. D gave compound **4 **and Fr. E gave compounds **5**, **6**, and **7**. Fr. F eluted with 25% ethyl acetate in *n*-hexane and rechromatographed with 25% ethyl acetate in *n*-hexane on silica gel column to obtain compound **8**. Fr. G was fractioned by column chromatography over silica gel using *n*-hexane:ethyl acetate mixtures (70:30 and 90:10) consecutively and compound **9** was obtained. Fr. H was submitted on a silica gel column using *n*-hexane:ethyl acetate (65:35) to yield compound **10** and the resulting fraction was chromatographed on silica gel column using *n*-hexane:ethyl acetate (90:10) to give compound **11**. Fr. I gave compound **12. **Compounds **1-4**, **6**, **8, 9, 11** and **13-14** were isolated by the same chromatographic methods in all species. Compounds **5**,** 10 **and** 12** were isolated only from dichloromethane fractions of roots from *F. bracteata, F. blancheana*, and *F. trachycarpa*, respectively. Compound **7** was isolated only from dichloromethane fractions of roots from both *F. trachycarpa* and *F. bracteata. *The yield of compounds from *Ferulago *species (%) are given at Table 2.

*Identification of isolated compounds*

**Figure 1:**
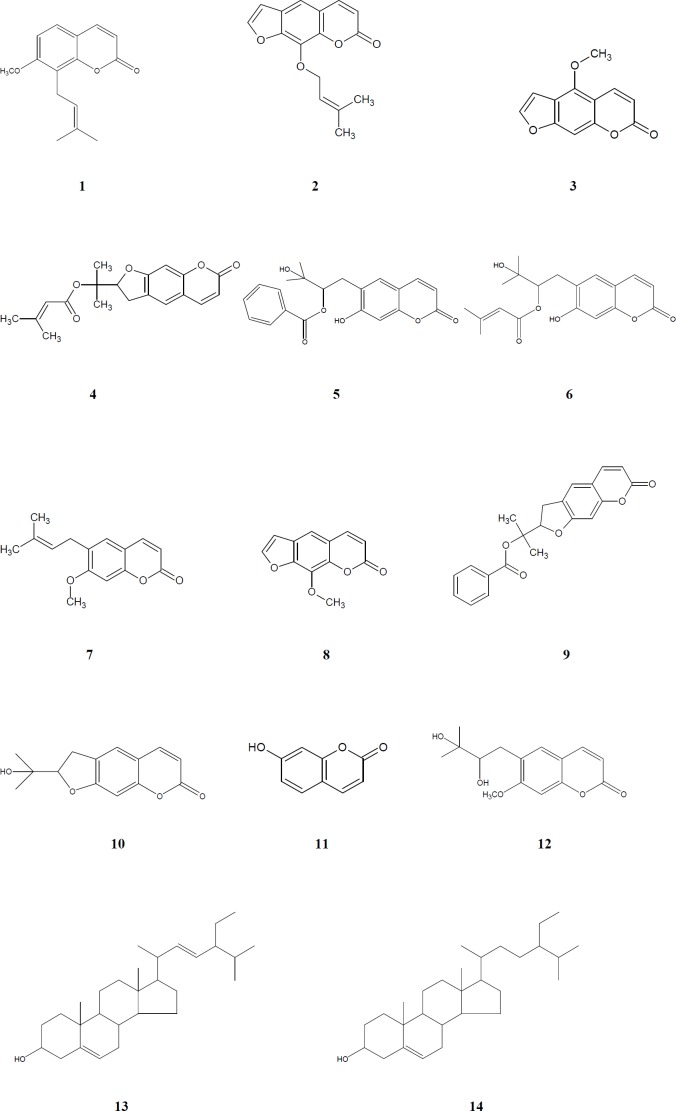
Chemical structures of compounds **1-14**

The structures of these isolated compounds were elucidated by means of detailed spectroscopic methods [NMR (Varian Mercury 400 MHz), ESI-MS (Waters Micromass ZQ Mass Spectrometer), HR-ESI-MS (Agilent 6530 Accurate-Mass Q-TOF LC/MS), UV (Thermo Scientific Multiskan GO Microplate and Cuvette Spectrophotometer), IR (Bruker VERTEX 70v FT-IR Spectrophotometer)]. Chemical structures of compounds **1-14** are given at Figure 1.

*Preparation of inoculum*

Firstly, standard strains at -80 °C were inoculated to Mueller Hinton Broth (MHB, Merck) culture medium for bacteria and Sabouraud Dextrose Broth (SDB, Oxoid) for yeast and after 24 h of incubation (at 37 °C for bacteria and at 25 °C for *Candida albicans*) passages were made on Mueller Hinton Agar (MHA, Merck) medium and Sabouraud Dextrose Agar (SDA, Oxoid) to obtain single colony culture. They were left for 24 h incubation to obtain sufficient reproduction. Isolated colonies from fresh overnight culture of these strains were inoculated into physiological saline solution to turbidity compared to that of 0.5 McFarland standards. Then, 0.1 mL of these bacterial suspensions were transferred into tubes containing 20 mL of MHB for bacteria and SDB for yeast. This bacterial suspension was used in experiments.

*Antimicrobial assay*

Studies of the extracts, fractions, and isolated compounds were performed due to the standard reference methods for bacteria *S. aureus* ATCC 29213, *E. coli* ATCC 25922, *P. aeruginosa* ATCC 27853, *B. subtilis* ATCC 6633 and for yeast *C. albicans* ATCC 10231. Minimum inhibitor concentration (MIC) was determined using the macrodilution broth method. The required concentrations of the compounds were dissolved in DMSO (2%). One milliliter of extract was added in the first tube for each extract to be tested. Then, two fold 8 serial dilutions were made to give concentrations ranging from 1000 to 7.81 µg/mL. After adding 1 mL bacterial suspensions to the tubes, they were left for 18-24 h incubation. As a-negative control, only the bacterial suspension was added into 9^th^ tube containing 1 mL of broth media. At the end of the incubation period, the assessments were evaluated according to the turbidity of the tubes. The tests were carried out according to the CLSI recommendations (37).

*Statistical analysis*

**Figure 2:**
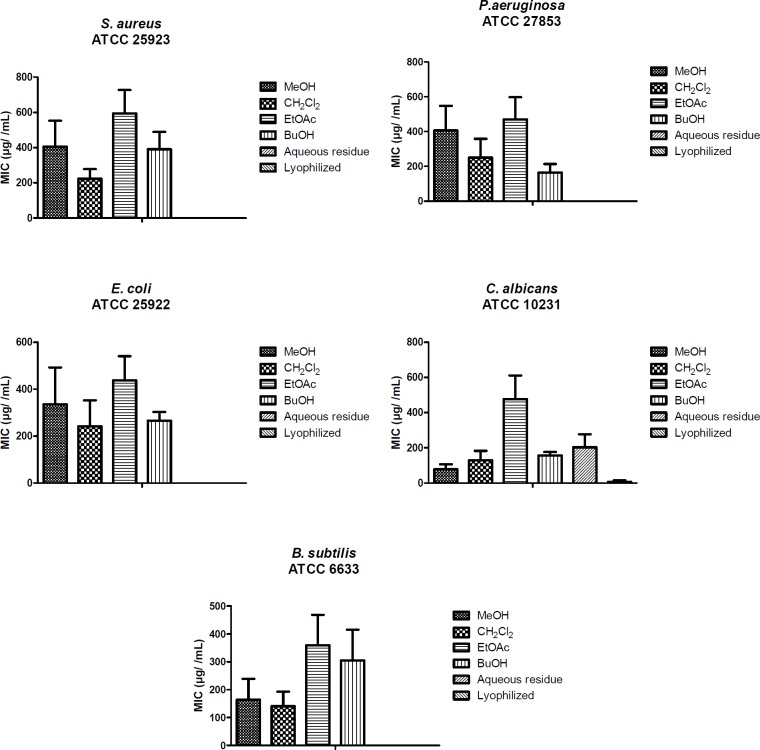
Statistical results of antimicrobial activity of all extracts and fractions against *S. aureus, E. coli,* *P. aeruginosa, C. albicans* and* B. subtilis, *respectively*.* *P* < 0.05 (*P* = 0.0004, 0.0012, 0.0023, 0.0079 and 0.0045, respectively)

All the results are expressed as mean ± SE and the differences between means were statistically analyzed using one-way analysis of ANOVA followed by Bonferroni’s complementary analysis, with *P* < 0.05 considered to indicate statistical significance (Figure 2).

## Results and Discussion

Methanol extracts of the aerial parts and roots of four *Ferulago *species were fractionated using solvents with different polarities (dichloromethane, ethyl acetate and *n*-butanol) and the obtained fractions were evaluated for their antimicrobial activities. The active dichloromethane extracts were subjected to column chromatography over silica gel and Sephadex LH-20. As the result, a coumarin, peucedanol-2′-benzoate (**5**) (31), together with eleven known ones, osthole (**1**) (38), imperatorin (**2**) (39), bergapten (**3**) (40), prantschimgin (**4**) (41), grandivitinol (**6**) (42), suberosin (**7**) (43), xanthotoxin (**8**) (40), felamidin (**9**) (44), marmesin (**10**) (45), umbelliferone (**11**) (46), ulopterol (**12**) (47) and a sterol mixture consisted of stigmasterol (**13**), *β*-sitosterol (**14**) (48) were isolated and identified. 

Osthole **(1)**. White powder, C_15_H_16_O_3_. ^13^C NMR (100 MHz, CDCl_3_) δ 161.34 (C-2), 112.85 (C-3), 143.80 (C-4), 126.7 (C-5), 107.37 (C-6), 160.21 (C-7), 117.84 (C-8), 152.78 (C-9), 112.95 (C-10), 21.91 (C-1’), 121.16 (C-2’), 132.54 (C-3’), 25.77 (C-4’), 17.91 (C-5’), 56.03 (OMe). ^1^H NMR (400 MHz, CDCl_3_) δ 3.91 (3H, s, OMe), 3.51 (2H, d, J = 7.2 Hz, H-1’), 5.22 (1H, m, H-2’), 1.66 (3H, s*, *H-4’), 1.83 (3H, s, H-5’), 6.20 (1H, d, J = 9.4 Hz, H-3), 7.60 (1H, d, J = 9.4 Hz, H-4), 7.27 (1H, d, J = 8.6 Hz, H-5), 6.82 (1H, d, J = 8.6 Hz, H-6). ESIMS m/z 245.31 [M+H]^+^. 

Imperatorin **(2)**. White powder, C_16_H_14_O_4_. ^13^C NMR (100 MHz, CDCl_3_) δ 159.82 (C-2), 114.20 (C-3), 145.34 (C-4), 114.12 (C-5), 125.72 (C-6), 147.83 (C-7), 130.56 (C-8), 143.21 (C-9), 116.39 (C-10), 146.43 (C-2’), 107.10 (C-3’), 69.38 (C-1″), 119.70 (C-2″), 139.12 (C-3″), 17.85 (C-4″), 25.49 (C-5″). ^1^H NMR (400 MHz, CDCl_3_) δ 6.29 (1H, d, J = 9.4 Hz, H-3), 7.73 (1H, d, J = 9.4 Hz, H-4), 7.29 (1H, s, H-5), 7.67 (1H, d, J = 2.1 Hz, H-2’), 6.78 (1H, d, J = 2.1 Hz, H-3’), 4.98 (2H, d, J = 7.0 Hz, H-1″), 5.56 1H, t, J = 7.0 Hz, H-2″), 1.79 (3H, s, H-4″, 5″). ESIMS m/z 271.29 [M+H]^+^. 

Bergapten **(3)**. White powder, C_12_H_8_O_4_. ^13^C NMR (100 MHz, CDCl_3_) δ 161.21 (C-2), 112.52 (C-3), 139.22 (C-4), 149.53 (C-5), 112.65 (C-6), 158.39 (C-7), 93.87 (C-8), 152.75 (C-9),106.43 (C-10), 144.76 (C-2’), 105.04 (C-3’), 60.04 (OMe). ^1^H NMR (400 MHz, CDCl_3_) 4.28 (3H, s, OMe), 6.26 (1H, d, J* =* 9.8 Hz, H-3), 8.17 (1H, d, J* =* 9.8 Hz, H-4), 7.15 (1H, s, H-8), 7.61 (1H, d, J* =* 2.1 Hz, H-2’ ), 7.05 (1H, bs, H-3’). ESIMS m/z 217.20 [M+H]^+^. 

Prantschimgin **(4). **Colourless crystals, C_19_H_20_O_5_. ^13^C NMR (100 MHz, CDCl_3_) δ 163.36 (C-2), 112.19 (C-3), 143.72 (C-4), 116.90 (C-5), 123.23 (C-6), 161.46 (C-7), 97.91 (C-8), 155.74 (C-9), 112.24 (C-10), 88.87 (C-2’), 29.57 (C-3’), 81.28 (C-1″), 22.29 (C-2″), 21.12 (C-3″), 165.85 (C-1”’), 124.57 (C-2”’), 156.47 (C-3”’), 20.66 (C-4”’), 27.40 (C-5”’). ^1^H NMR (400 MHz, CDCl_3_) δ 6.22 (1H, d, J = 9.8 Hz, H-3), 7.61 (1H, d, J = 9.8 Hz, H-4), 7.22 (1H, s, H-5), 6.75 (1H, s, H-8), 5.14 (1H, dd, J* = *8.0, 8.8 Hz, H-2’), 3.23 (2H, m, H-3’), 1.60 (3H, s, H-2”), 1.54 (3H, s, H-3”), 5.56 (1H, s, H-2”’), 2.10 (3H, s, H-4”’), 1.86 (3H, s, H-5”’). ESIMS m/z 329.14 [M+H]^+^. 

Peucedanol-2’-benzoate (**5**). White powder, C_21_H_20_O_6_. IR ν_max_ (KBr) cm^-1^: 1702, 1623, 1565. UV λ_max_ (CH_2_Cl_2_) nm (log *ɛ*): 350 (4.20). ^13^C NMR (100 MHz, CDCl_3_) δ 161.38 (C-2), 112.37 (C-3), 143.62 (C-4), 123.22 (C-5), 124.55 (C-6), 163.49 (C-7), 98.01 (C-8), 155.84 (C-9), 112.71 (C-10), 29.67 (C-1’), 89.12 (C-2’), 82.93 (C-3’), 22.16 (C-4’), 21.38 (C-5’), 165.40 (C-1”), 131.04 (C-2”), 129.39 (C-3”, 7”), 128.27 (C-4”, 6”), 132.86 (C-5”). ^1^H NMR (400 MHz, CDCl_3_) δ 6.26 (1H, d, J = 9.4 Hz, H-3), 7.64 (1H, d, J = 9.4 Hz, H-4), 7.28 (1H, s, H-5), 6.80 (1H, s, H-8). 3.38 (2H, m, H-1’), 5.16 (1H, dd, J = 9.2/7.3 Hz, H-2’), 1.72 (3H, s*, *H-4’), 1.71 (3H, s, H-5’), 7.73 (1H, m, H-3”, 7”), 7.32 (1H, m, H-4”, 6”), 7.51 (1H, m, H-5”). HRESIMS at m/z 367.1999 [M-H]^+^. 

Grandivitinol **(6)**. White powder, C_19_H_22_O_6_. ^13^C NMR (100 MHz, CDCl_3_) δ 161.41 (C-2), 112.24 (C-3), 143.65 (C-4), 123.22 (C-5), 124.54 (C-6), 163.43 (C-7), 97.93 (C-8), 155.78 (C-9), 112.68 (C-10), 29.58 (C-1’), 88.85 (C-2’), 81.29 (C-3’), 22.29 (C-4’), 21.25 (C-5’), 165.84 (C-1”), 116.92 (C-2”), 156.60 (C-3”), 27.37 (C-4”), 20.06 (C-5”). ^1^H NMR (400 MHz, CDCl_3_) δ 6.22 (1H, d, J = 9.4 Hz, H-3), 7.61 (1H, d, J = 9.4 Hz, H-4), 7.22 (1H, s, H-5), 6.75 (1H, s, H-8), 3.24 (2H, m, H-1’), 5.15 (1H, dd, J = 9.3/7.9 Hz, H-2’), 1.61 (3H, s, H-4’), 1.55 (3H, s, H-5’), 5.57 (1H, t, J = 1.2 Hz, H-2’’), 1.87 (3H, d, J = 1.2 Hz, H-4’’), 2.11 (3H, d, J = 1.2 Hz, H-5’’). ESIMS m/z 347.10 [M+H]^+^.

Suberosin **(7)**. Colourless crystal, C_15_H_16_O_3_. ^13^C NMR (100 MHz, CDCl_3_) δ 161.53 (C-2), 112.76 (C-3), 143.64 (C-4), 127.4 (C-5), 127.49 (C-6), 160.65 (C-7), 98.5 (C-8), 154.48 (C-9), 111.90 (C-10), 27.79 (C-1’), 121.35 (C-2’), 132.67 (C-3’), 25.82 (C-4’), 17.77 (C-5’), 55.86 (OMe). ^1^H NMR (400 MHz, CDCl_3_) δ 3.91 (3H, s, OMe), 3.32 (2H, d, J = 7.3 Hz, H-1’), 5.29 (1H, tt, J = 7.3/1.3 Hz, H-2’), 1.78 (3H, s*, *H-4’), 1.71 (3H, s, H-5’), 6.24 (1H, d, J = 9.4 Hz, H-3), 7.63 (1H, d, J = 9.4 Hz, H-4), 7.19 (1H, s, H-5), 6.78 (1H, s, H-6). ESIMS m/z 245.31 [M+H]^+^.

**Table 1:** Amounts of the powdered plants and obtained extracts and fractions

**Species**	**Used parts**	**Powdered (g)**	**MeOH** **(g)**	**CH** **2** **Cl** **2** **(g)**	**EtOAc (g)**	**BuOH** **(g)**	**Aqueous residue (g)**	**Lyophilized Aqueous (g)**
*F. blancheana*	root	750	86.62	28.52	2.32	12.24	23.35	5.78
	aerial part	50	3.22	1.89	0.46	0.57	0.39	1.78
*F. pachyloba*	root	600	83.25	23.63	1.53	13.13	21.29	4.98
	aerial part	50	3.32	1.78	0.45	0.59	0.45	2.01
*F. trachycarpa*	root	450	86.77	26.29	2.41	13.55	22.08	4.76
	aerial part	50	3.41	1.67	0.50	0.61	0.55	1.67
*F. bracteata*	root	450	60.94	17.96	2.44	14.98	13.98	3.99
	aerial part	50	3.65	1.55	0.61	0.59	0.61	1.88

**Table 2:** The yield of compounds from Ferulago species (%).

**Species**	Compounds												
	**1**	**2**	**3**	**4**	**5**	**6**	**7**	**8**	**9**	**10**	**11**	**12**	**13- 14**
*F. pachyloba*	0.0366	0.0208	0.0216	0.0666	-	0.0250	-	0.0183	0.0541	-	0.0408	-	0.0733
*F. trachycarpa*	0.0314	0.0178	0.0185	0.0571	-	0.0214	0.044	0.0157	0.0464	-	0.0350	0.0457	0.0628
*F. bracteata*	0.0488	0.0166	0.0155	0.0222	0.0711	0.0210	0.7333	0.0091	0.0348	-	0.0277	-	0.0466
*F. blancheana*	0.0295	0.0162	0.0173	0.0533	-	0.0200	-	0.0146	0.0433	0.0146	0.0326	-	0.0586

**Table 3:** Antimicrobial activity (MIC values µg/mL) of extracts, fractions and compounds and reference antibiotics

**Samples**	**Extracts and Fractions**	***S. aureus*** **ATCC 25923**	***E. coli*** **ATCC 25922**	***P. aeruginosa*** **ATCC 27853**	***B. subtilis*** **ATCC 6633**	***C. albicans*** **ATCC 10231**
*F. blancheana* aerial part	MeOH	500	500	250	62.5	125
	CH2Cl2	250	250	125	125	125
	EtOAc	-	-	-	-	-
	BuOH	125	125	62.5	250	125
	Aqueous residue	-	-	-	-	250
	Lyophilized aqueous	-	-	-	-	62.5
*F. blancheana* root	MeOH	125	-	250	125	62.5
	CH2Cl2	500	1000	1000	500	500
	EtOAc	500	500	500	250	500
	BuOH	1000	500	500	1000	125
	Aqueous residue	-	-	-	-	500
	Lyophilized aqueous	-	-	-	-	-
*F. pachyloba* aerial part	MeOH	-	-	-	-	-
	CH2Cl2	125	125	125	62.5	31.25
	EtOAc	1000	500	1000	500	500
	BuOH	250	250	125	500	125
	Aqueous residue	-	-	-	-	-
	Lyophilized aqueous	-	-	-	-	-
*F. pachyloba* root	MeOH	-	-	-	-	-
	CH2Cl2	125	125	250	62.5	62.5
	EtOAc	1000	500	500	250	1000
	BuOH	500	250	125	125	250
	Aqueous residue	-	-	-	-	-
	Lyophilized aqueous	-	-	-	-	-
*F. trachycarpa* aerial part	MeOH	500	62.5	250	500	250
	CH2Cl2	250	125	125	125	125
	EtOAc	1000	1000	1000	1000	1000
	BuOH	250	250	125	125	125
	Aqueous residue	-	-	-	-	250
	Lyophilized aqueous	-	-	-	-	-
	MeOH	125	1000	1000	62.5	62.5
	CH2Cl2	62.5	62.5	125	62.5	62.5
	EtOAc	250	250	250	250	500
*F. trachycarpa* root	BuOH	500	250	125	250	125
	Aqueous residue	-	-	-	-	125
	Lyophilized aqueous	-	-	-	-	-
	MeOH	1000	1000	500	500	62.5
	CH2Cl2	250	125	125	125	62.5
	EtOAc	500	500	250	500	250
*F. bracteata* aerial part	BuOH	250	250	125	62.5	250
	Aqueous residue	-	-	-	-	-
	Lyophilized aqueous	-	-	-	-	-
	MeOH	1000	125	1000	62.5	62.5
	CH2Cl2	125	125	125	62.5	62.5
	EtOAc	500	250	250	125	62.5
*F. bracteata* root	BuOH	250	250	125	125	125
	Aqueous residue	-	-	-	-	500
	Lyophilized aqueous	-	-	-	-	-
Osthole		500	500	250	500	500
Felamidin		500	250	250	500	500
Grandivitinol		1000	500	250	500	125
Umbelliferone		250	500	250	500	125
Prantschimgin		500	250	250	250	31.25
Ulopterol		500	500	500	250	250
Marmesin		500	250	250	250	250
Mixture of Stigmasterol and *β*-sitosterol		1000	250	500	1000	250
Streptomycin		6.25	25	25	25	
Ciprofloxacin		<0.78	6.25	6.25	<0.78	
Ketaconazole						25
Chloromycin		4	16	8	4	-
Miconazole		>100	>100	>100	-	3

**Figure 3:**
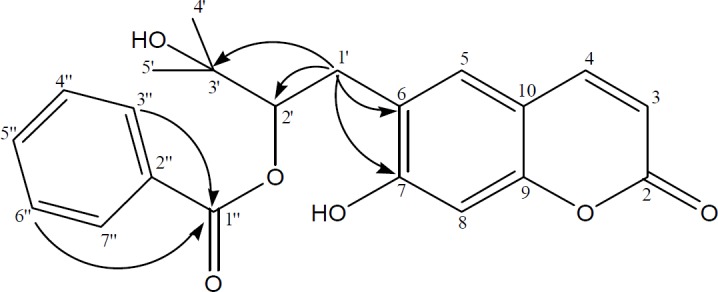
Significant HMBC (→) correlations of compound **5**

Xanthotoxin **(8)**. White powder, C_12_H_8_O_4_. ^13^C NMR (100 MHz, CDCl_3_) δ 160.8 (C-2), 114.5 (C-3), 143.60 (C-4), 112.90 (C-5), 126.4 (C-6), 147.8 (C-7), 132.61 (C-8), 143.90 (C-9), 116.51 (C-10), 146.7 (C-2’), 106.79 (C-3’), 61.20 (OMe-8). ^1^H NMR (400 MHz, CDCl_3_) δ 4.31 (3H, s, OMe), 6.35 (1H, d, J = 9.8 Hz, H-3), 7.77 (1H, d, J = 9.8 Hz, H-4), 7.35 (1H, s, H-5), 7.68 (1H, d, J = 2.4 Hz, H-2’), 6.83 (1H, d, J = 2.4 Hz, H-3’). ESIMS m/z 217.19 [M+H]^+^.

Felamidin **(9)**. Colourless crystal, C_21_H_18_O_5_. ^13^C-NMR (100 MHz, CDCl_3_): δ 161.0 (C-2), 112.20 (C-3), 143.60 (C-4), 123.20 (C-5), 124.50 (C-6), 163.40 (C-7), 97.90 (C-8), 155.8 (C-9), 112.7 (C-10), 89.10 (C-2’), 29.60 (C-3’), 82.90 (C-1’’), 22.1 (C-2″), 21.4 (C-3″), 165.30 (C-1’’’), 131.00 (C-2’’’), 128.2 (C-3’’’, 7’’’), 129.4 (C-4’’’, 6’’’), 132.8 (C-5’’’). ^1^H-NMR (400 MHz, CDCl_3_) δ 6.34 (1H, d, J = 9.57 Hz, H-3), 7.73 (1H, d, J = 9.57 Hz, H-4), 7.36 (1H, s, H-5), 6.88 (1H, s, H-8), 5.24 (1H, m, H-2’), 3.40~3.50 (2H, m, H-3’), 1.71 (3H, s, H-2″), 1.69 (3H, s, H-3″), 7.83 (2H, m, H-3’’’, 7’’’), 7.36 (2H, m, H-4’’’, 6’’’), 7.56 (1H, m, H-5’’’). ESIMS m/z 351.22 [M+H]^+^. 

Marmesin **(10)**. White powder, C_14_H_14_O_4_. ^13^C NMR (100 MHz, CDCl_3_) δ 161.52 (C-2), 112.10 (C-3), 143.75 (C-4), 123.41 (C-5), 125.17 (C-6), 163.22 (C-7), 97.84 (C-8), 155.57 (C-9), 112.70 (C-10), 91.17 (C-2’), 29.45 (C-3’), 71.60 (C-1’’), 26.07 (C-2’’), 24.35 (C-3’’). ^1^H NMR (400 MHz, CDCl_3_) δ 6.18 (1H, d, J = 9.4 Hz, H-3), 7.58 (1H, d, J = 9.4 Hz, H-4), 7.21 (1H, s, H-5), 6.96 (1H, s, H-8), 4.74 (1H, t, J* = *8.9 Hz, H-2’), 3.22 (2H, m, H-3’), 1.37 (3H, s, H-2’’), 1.24 (3H, s, H-3’’). ESIMS m/z 247.21 [M+H]^+^.

Umbelliferone **(11)**. Creamy powder, C_9_H_6_O_3_. ^13^C NMR (100 MHz, CDCl_3_) δ 162.34 (C-2), 110.87 (C-3), 144.54 (C-4), 129.37 (C-5), 113.11 (C-6), 161.74 (C-7), 102.02 (C-8), 155.82 (C-9), 111.71 (C-10). ^1^H NMR (400 MHz, CDCl_3_) δ 6.14 (1H, d, J* =* 9.3 Hz, H-3), 7.78 (1H, d, J* =* 9.3 Hz, H-4), 7.37 (1H, d, J* =* 8.7 Hz, H-5), 6.72 (1H, d, J* =* 8.4 Hz, H-6), 6.63 (1H, s, H-8). ESIMS m/z 163.24 [M+H]^+^. 

Ulopterol **(12)**. White powder, C_15_H_18_O_5_. ^13^C NMR (100 MHz, CDCl_3_) δ 161.18 (C-2), 112.52 (C-3), 145.07 (C-4), 130.14 (C-5), 126.57 (C-6), 161.22 (C-7), 98.89 (C-8), 154.41 (C-9), 111.87 (C-10), 31.64 (C-1’), 76.87 (C-2’), 72.35 (C-3’), 26.54 (C-4’), 25.14 (C-5’), 56.59 (OMe). ^1^H NMR (400 MHz, CDCl_3_) δ 6.24 (1H, d, J = 9.4 Hz, H-3), 7.94 (1H, d, J = 9.4 Hz, H-4), 7.46 (1H, s, H-5), 6.96 (1H, s, H-8), 2.96 (1H, d, J *= *14.0 Hz, H-1’a), 2.32 (1H, dd, J* = *14.0/10.4 Hz, H-1’b), 3.38 (1H, m, H-2’), 1.11 (3H, s, H-4’), 1.09 (3H, s, H-5’), 3.85 (3H, s, OMe); ESIMS m/z 279.09 [M+H]^+^.

Stigmasterol **(13)**. White powder, C_29_H_48_O. ^13^C NMR (100 MHz, CDCl_3_) δ 37.26 (C-1), 28.92 (C-2), 71.83 (C-3), 42.29 (C-4), 140.75 (C-5), 121.72 (C-6), 31.65 (C-7), 31.9 (C-8), 50.14 (C-9), 36.51 (C-10), 24.37 (C-11), 39.69 (C-12), 42.33 (C-13), 56.78 (C-14), 25.41 (C-15), 29.7 (C-16), 55.97 (C-17), 12.3 (C-18), 19.4 (C-19), 40.49 (C-20), 21.08 (C-21), 138.32 (C-22), 129.29 (C-23), 51.24 (C-24), 31.91 (C-25), 19.0 (C-26), 19.05 (C-27), 29.7 (C-28), 11.9 (C-29). ^1^H NMR (400 MHz, CDCl_3_) δ 3.55 (1H, m, H-3), 5.36 (1H, bd, J = 5.16 Hz, H-6), 0.7 (3 H, s, H-18), 1.03 (3H, s, H-19), 5.17 (1H, dd, J* = *15.1/8.6 Hz, H-22), 5.03 (1H, dd, J *= *15.1/8.6 Hz, H-23), 0.82 (3H, d, J* = *7.1 Hz, H-26), 0.81 (3H, d, J* = *7.0 Hz, H-27).

*β*-Sitosterol **(14)**. White powder, C_29_H_50_O. ^13^C NMR (100 MHz, CDCl_3_) δ 37.26 (C-1), 31.65 (C-2), 71.83 (C-3), 42.29 (C-4), 140.75 (C-5), 121.72 (C-6), 31.65 (C-7), 31.9 (C-8), 50.14 (C-9), 36.51 (C-10), 21.22 (C-11), 39.78 (C-12), 42.22 (C-13), 56.87 (C-14), 25.31 (C-15), 28.25 (C-16), 56.07 (C-17), 12.0 (C-18), 19.4 (C-19), 36.15 (C-20), 18.9 (C-21), 33.95 (C-22), 26.09 (C-23), 45.85 (C-24), 29.16 (C-25), 19.8 (C-26), 19.4 (C-27), 23.07 (C-28), 12.1 (C-29). ^1^H NMR (400 MHz, CDCl_3_) δ 3.55 (1H, m, H-3), 5.36 (1H, bd, J = 5.1 Hz, H-6), 0.71 (3H, s, H-18), 1.03 (3H, s, H-19), 0.94 (3H, d, J* = *6.6 Hz, H-21), 0.82 (3H, d, J *= *7.1 Hz, H-26), 0.81 (3H, d, J* = *7.0 Hz, H-27).

Peucedanol-2′-benzoate (**5**) was isolated as a white powder with the molecular formula of C_21_H_20_O_6_ as determined by the HR-ESI-MS at *m/z* 367.1999 [M-H]^+^ (Calcd for C_21_H_19_O_6_ 367.1181). The IR spectrum of **5** showed absorption bands for C=O groups (1702 cm^-1^) and -CH=CH- bonds (1623, 1565 cm^-1^). The ^1^H NMR spectrum of compound **5** displayed two AB type system protons at δ_H_ 6.26 and 7.64 (each 1H, d, J = 9.4 Hz) which was attributed to the H-3 and H-4 protons of the coumarin nucleus. The two single aromatic proton signals at δ_H_ 7.28 and 6.80 were assigned to H-5 and H-8 protons. The ^13^C NMR spectrum revealed the presence of 9 carbons resonances including four methine [δ_C_ 112.37 (C-3), 143.62 (C-4), 123.22 (C-5), 98.01 (C-8)], three oxygenated quaternary [δ_C_ 161.38 (C-2), 163.49 (C-7), 155.84 (C-9)] and two non-oxygenated quaternary carbons [δ_C_ 124.55 (C-6), 112.71 (C-10)] for coumarin skeleton. Two tertiary methyls at δ_H_ 1.72 (3H, s, H-4′), 1.71 (3H, *s*, H-5′) and at δ_C_ 22.16 (C-4′), 21.38 (C-5′) with the hydroxyl group; an oxygenated methine at δ_H_ 5.16 (1H, dd, J = 9.2/7.3 Hz, H-2′) and at δ_C_ 89.12 (C-2′); and a methylene at δ_H_ 3.38 (2H, m, H-1′) and at δ_C_ 29.67 (C-1′) confirmed the 2′,3′-dihydroxy-3′-methyl butyl moiety. HMBC correlation (Figure 3) between H-1′ (δ_H_ 3.38) and C-6 (δ_C_ 124.55) suggested that it was attached to C-6 position. Characteristic signals of a benzoyl moiety were also exhibited, including a pair of 2H at δ_H_ 7.73 (H-3′′, H-7′′) and 7.32 (H-4′′, H-6′′) and 1H at 7.51 (H-5′′) in the ^1^H NMR spectrum and aromatic carbons at δ_C_ 131.04 (C-2′′), 129.39 (C-3′′, C-7′′), 128.27 (C-4′′, C-6′′), 132.86 (C-5′′) with a carbonyl carbon at δ_C_ 165.40 (C-1′′) in the ^13^C NMR spectrum. The linkage of the benzoyl group to the 2′,3′-dihydroxy-3′-methyl butyl moiety was deduced from the downfield shifted signal of H-2′ (*δ*_H_ 5.16) and C-2′ (*δ*_C_ 89.12). Thus, the structure of the compound **5** was characterized as peucedanol-2′-benzoate.

The antimicrobial activities of the extracts, fractions, and isolated compounds have been given in Table 3 as MIC values. These compounds showed a broad range of (31.25-1000 µg/mL) antimicrobial activity. Among the lyophilized aqueous extracts only aerial parts of *F. blancheana* and also, among the aqueous residue fractions only aerial parts and roots of *F. blancheana*, *F. trachycarpa*, and roots of *F. bracteata* showed activity against *C. albicans. *Among the microorganisms *E. coli* was found to be least affected from extracts, fractions, and pure compounds. Among the prepared extracts, fractions and the obtained pure compounds the best effect against *S. aureus, E. coli, P. aeruginosa, B. subtilis*, and *C. albicans* were determined with methanol extract and *n*-butanol fractions of aerial parts, methanol extract of roots from *F. blancheana *(62.5 µg/mL); dichloromethane fraction of roots from *F. trachycarpa* (62.5 µg/mL); dichloromethane fraction of aerial parts and roots from *F. pachyloba* (31.25, 62.5 µg/mL); methanol extracts of roots and aerial parts and dichloromethane fraction of roots from *F. trachycarpa* (62.5 µg/mL); methanol extracts and dichloromethane fractions of roots and aerial parts, ethyl acetate fraction of roots, *n*-butanol fraction of aerial parts from *F. bracteata *(62.5 µg/mL), and isolated compound prantschimgin (31.25 µg/mL). In particular, prantschimgin had a remarkable activity against *C. albicans*, which is responsible for severe infections and is very often resistant to conventional antifungal drugs. 

Our results were similar to previous studies of related coumarins. Karunai *et al.* (2012) found that ulopterol showed appreciable antimicrobial activity against some Gram negative and Gram positive microorganisms (49). Ojala *et al.* (2000) indicated that umbelliferone showed antibacterial activity against *P. aeruginosa*, bacteriostatic activity against *E. coli*; however, it did not show any activity against *S. aureus, B. subtilis*, and *C. albicans* (50). Golfakhrabadi *et al.* (2016) reported that prantschimgin had antimicrobial activity against *S. aureus, P. aeruginosa, C. albicans,* and also no activity against *E. coli* (32). Basile *et al.* (2009) showed that felamidin exposed antimicrobial activity against *S. aureus* and *P. aeruginosa *(8). It was reported that petroleum ether extracts of *F. asparagifolia, F. aucheri*, and chloroform extract of *F. humilis* which were collected from Aegean division of Turkey, did not show any significant activity by disc diffusion method against the tested microorganisms (49). Bostanlık *et al.* (2015) found that the extracts of *Ferulago sandrasica* Peşmen and Quezel and *Ferulago mughlae* Peşmen had antimicrobial activity against *S. aureus* ATCC 25923, but no activity against *E. coli* ATCC 25922, *P. aeruginosa* ATCC 27853, and *B. subtilis* ATCC 6633 (51). Differences come from the difference of the compounds and their quantities among species. It is important to find a species that has a wide range of antimicrobial activity in the genus. Nowadays, due to the rapid increases in resistance to antibiotics, researches are shifting to create new combinations of active compounds derived from natural products. Besides, the consumers prefer foods with natural preservatives. As we mentioned before, these species except *F. trachycarpa* are endemic and this is the first report of their antimicrobial activity. We think that results of our study will contribute to the investigations in new antibiotic combinations or food preservatives. Therefore, based on our results and regarding the results of our colleagues, it seems that the biological activity assessed and sighted in the current study could be related to the synergistic effect of the different compounds included in these species. It is hoped that the research and development studies on the antimicrobial effects of plant-derived compounds in relation to the use of current technological conditions, will broaden the scope of the solution field.

In conclusion, among the isolated compounds prantschimgin has emerged as new target for antimicrobial diseases. Therefore, we can conclude that prantschimgin can be used in antimicrobial diseases and may represent an herbal alternative to synthetic drugs.
